# Human Neuronal Modeling of DLG4-Related Synaptopathy Reveals Network Dysfunction and Partial Rescue by Omega-3 Fatty Acid Docosahexaenoic Acid (DHA)

**DOI:** 10.3390/cells15181688

**Published:** 2026-09-17

**Authors:** Gerardo Medina, Lilia A. Rodriguez-Alcocer, Stephen Baird, Alex E. MacKenzie

**Affiliations:** 1 Children’s Hospital of Eastern Ontario Research Institute, Ottawa, ON K1H 8L1, Canada; lrodriguezalcocer@cheo.on.ca (L.A.R.-A.); s.d.baird@gmail.com (S.B.); 2 Department of Cellular and Molecular Medicine, Faculty of Medicine, University of Ottawa, Ottawa, ON K1H 8M5, Canada; 3 Eric Poulin Center for Neuromuscular Disease, Faculty of Medicine, University of Ottawa, Ottawa, ON K1H 8M5, Canada

**Keywords:** DLG4, SHINE syndrome, PSD-95, induced pluripotent stem cells (iPSCs), docosahexaenoic acid (DHA), omega-3 fatty acids, neuronal network activity, synaptopathy

## Abstract

**Highlights:**

**What are the main findings?**

- SHINE iPSC-derived neurons with DLG4 haploinsufficiency exhibit reduced PSD-95, altered neuronal morphology, and impaired neuronal network activity.
- DHA treatment increases PSD-95 abundance and improves neuronal firing, burst dynamics, and network synchrony in SHINE neurons.

**What are the implications of the main findings?**

- Human iPSC-derived neurons provide a disease-relevant model for investigating molecular, structural, and network dysfunction associated with DLG4-related synaptopathy.
- The response to DHA supports further investigation of lipid-based approaches to modify synaptic and network dysfunction in SHINE syndrome.

**Abstract:**

DLG4-related synaptopathy (SHINE syndrome) is a rare neurodevelopmental disorder caused by pathogenic variants in *DLG4*, which encodes the postsynaptic scaffolding protein (PSD)-95, a central organizer of excitatory synapses. However, the cellular and network-level consequences of DLG4 haploinsufficiency in human neurons remain incompletely understood, and disease-targeted therapeutic strategies are lacking. Here, we used human induced neurons derived from an individual with SHINE syndrome to investigate the effects of PSD-95 deficiency on synaptic protein expression, neuronal morphology, and neuronal network activity. SHINE neurons exhibited reduced PSD-95 levels, impaired neurite morphology, reduced firing rate and burst frequency, prolonged burst duration, and reduced network synchrony. Treatment with the omega-3 (ω-3) polyunsaturated fatty acid docosahexaenoic acid (DHA) significantly increased PSD-95 protein levels and improved multiple parameters of neuronal network activity. DHA exposure was also associated with trends toward increased extracellular signal-regulated kinase and cAMP response element-binding protein phosphorylation. Together, these findings show that patient-derived SHINE neurons exhibit altered postsynaptic organization, neuronal morphology, and network function and provide evidence that DHA may partially restore synaptic and network function in this disorder.

## 1. Introduction

Neurotransmitter release from presynaptic terminals triggers signal transduction in postsynaptic neurons throughout the brain. At excitatory synapses, this process is organized by the highly structured postsynaptic density (PSD), a complex that anchors neurotransmitter receptors, scaffolding proteins, and signaling molecules required for synaptic transmission and plasticity [1,2]. Among the key components of this structure is postsynaptic density protein-95 (PSD-95), a scaffolding protein encoded by the *DLG4* gene. PSD-95 plays a central role in the assembly and stabilization of excitatory synapses by linking glutamate receptors, including N-methyl-D-aspartate (NMDA) receptors and α-amino-3-hydroxy-5-methyl-4-isoxazolepropionic acid (AMPA) receptors, to intracellular signaling pathways and cytoskeletal elements [1,3,4]. Through these interactions, PSD-95 contributes to synaptic maturation, receptor clustering, and activity-dependent synaptic plasticity.

Alterations in PSD-95 expression or function can therefore have important consequences for neuronal connectivity and circuit function [5]. Experimental studies have shown that PSD-95 regulates the stabilization of AMPA receptors at synapses and contributes to the maturation of excitatory synaptic contacts [4,5,6,7]. Disruption of PSD-95 scaffolding has been associated with impaired dendritic spine development, altered synaptic architecture, and abnormalities in synaptic transmission [6,7,8]. Dendritic development provides the structural substrate for postsynaptic organization; changes in dendrite-associated proteins such as microtubule-associated protein 2 (MAP2) may provide an additional indication of altered neuronal structural maturation [9]. Because these processes are critical during neurodevelopment, changes in PSD-95 abundance or localization may lead to persistent alterations in neuronal network organization. Disruptions in PSD-95-dependent synaptic organization may therefore influence emergent neuronal network dynamics, including firing rate, burst activity, and synchrony.

Disabling mutations in a single *DLG4* allele cause a rare neurodevelopmental disorder commonly referred to as DLG4-related synaptopathy or SHINE syndrome (Sleep disturbances, Hypotonia, Intellectual disability, Neurological disorders, and Epilepsy) [10,11,12]. Individuals with SHINE syndrome typically present with developmental delay, intellectual disability, epilepsy, hypotonia, and features of autism spectrum disorder (ASD) [10,13,14]. These clinical manifestations are thought to arise from impaired postsynaptic signaling and disrupted excitatory synaptic plasticity during critical stages of brain development [11]. Although human genetic studies have established the causal role of *DLG4* mutations in this disorder, the cellular mechanisms through which PSD-95 deficiency alters synaptic organization and neuronal network activity remain poorly understood.

Given that most DLG4-related synaptopathy variants do not produce functional PSD-95, interventions which increase PSD-95 levels represent a viable therapeutic strategy. This could potentially be achieved by increasing expression from the unaffected *DLG4* allele, by stabilizing PSD-95 protein and increasing its half-life, or by engaging pathways that enhance postsynaptic scaffold abundance. At present, however, there are no targeted pharmacological treatments for this disorder.

To identify candidate compounds capable of increasing PSD-95, we conducted a targeted neuronal screen of 18 agents previously reported to modulate PSD-95 expression or synaptic protein abundance. PSD-95 immunofluorescence intensity following 24 h treatment was used as the screening readout, and compounds were ranked according to the magnitude of change relative to vehicle-treated controls. The five highest-ranking candidates are shown in Figure S1. Among the compounds screened, docosahexaenoic acid (DHA), a long-chain omega-3 polyunsaturated fatty acid, produced the largest increase in PSD-95 immunofluorescence intensity. DHA is highly enriched in neuronal membranes and contributes to membrane fluidity, receptor organization, and intracellular signaling processes [15,16,17]. Experimental studies have also shown that DHA availability can influence neurite outgrowth, synaptogenesis, and the expression of synaptic proteins, including PSD-95 [18,19,20]. Thus, while DHA may have broader effects on neuronal membrane composition and synaptic stability, its relevance to DLG4-related synaptopathy may be primarily based on its potential to increase the postsynaptic scaffold protein that is deficient in this disorder.

Whether DHA can increase PSD-95 levels and improve neuronal network activity in the context of human DLG4 haploinsufficiency remains unknown. Here, we used human induced pluripotent stem cell (iPSC)-derived neurons from an individual with DLG4-related synaptopathy to investigate the cellular consequences of PSD-95 deficiency and to determine whether DHA supplementation can modify synaptic protein expression and neuronal network function. We found that SHINE neurons exhibit reduced PSD-95 expression, altered neuronal morphology, and impaired network activity. DHA treatment increased PSD-95 abundance and partially improved neuronal firing and network synchrony, supporting DHA as a candidate synaptic modifier in DLG4-related synaptopathy.

## 2. Materials and Methods

### 2.1. Human iPSC Lines

The iPSCs were derived from an individual diagnosed with SHINE syndrome and an age-matched unaffected control individual. The male SHINE patient (CH1371), who was 12 years old at the time of cell line derivation (year of birth: 2007), has a *DLG4* (c.455delG; p.G152Afs*12) variant. The single-nucleotide frameshift deletion at position 152 (glycine) results in a premature stop codon 12 amino acids later and a truncated, non-functional protein (normally the protein is 724 AA), given the likelihood of nonsense-mediated decay (NMD); likely a pathogenic haploinsufficiency. The patient has severe motor delay, is nonverbal, has ASD, attention-deficit/hyperactivity disorder (ADHD), hypotonia, ataxia, apraxia, insomnia, and manifests focal, focal to bilateral, and tonic seizures. More clinical information can be found in Table 4 of Ref [14] (patient 3).

A single wild-type (WT) control iPSC line (GM0323; male; year of birth: 2008), derived from fibroblasts and reprogrammed using ReproRNA-OKSGM RNA transfection in the presence of B18R, was used as a comparator. The control line had no pathogenic variants in *DLG4*. Both lines were differentiated into forebrain neurons using the same differentiation protocol and experimental conditions.

Immunofluorescence and Western blot experiments were performed in three independent experimental runs conducted on separate occasions using independently prepared neuronal cultures derived from the same SHINE and WT iPSC lines. For these assays, each independent experimental run was considered a biological replicate (*n* = 3). Microelectrode array (MEA) analysis was performed separately as a single longitudinal experiment using one neuronal differentiation batch and one MEA plate containing five replicate wells per condition. The same MEA wells were recorded at day in vitro (DIV) 44 and again at DIV45 following treatment.

### 2.2. Generation and Differentiation of Human iPSC-Derived Neurons

iPSCs derived from an individual with SHINE syndrome and an unaffected control were maintained under feeder-free conditions in mTeSR™ Plus medium (STEMCELL Technologies, Vancouver, BC, Canada, Catalog No. 100-0276) at 37 °C in a humidified incubator with 5% CO_2_. Culture medium was replaced daily, and colonies were passaged using standard enzymatic dissociation methods when reaching appropriate confluence.

Neural induction was performed using the STEMdiff™ SMADi Neural Induction Kit (STEMCELL Technologies, Catalog No. 08581), which directs neural lineage commitment through dual SMAD inhibition. Induction medium was prepared according to the manufacturer’s instructions and replaced daily until neural progenitor cell (NPC) morphology was established. NPCs were subsequently expanded in STEMdiff™ Neural Progenitor Medium (STEMCELL Technologies, Catalog No. 05833) with daily medium changes and passaged using Accutase (STEMCELL Technologies, Catalog No. 07922). Cells were expanded for a minimum of four passages before terminal differentiation.

For neuronal differentiation, NPCs were plated onto matrix-coated plates at a density of 4.0 × 10^4^ cells/cm^2^ and cultured using the STEMdiff™ Forebrain Neuron Differentiation Kit (STEMCELL Technologies, Catalog No. 08600) for 7 days with daily medium changes. Plates were coated with 15 µg/mL Poly-L-ornithine solution (PLO; Sigma, St. Louis, MO, USA, Catalog No. P4957-50ML) and 5 µg/mL laminin from Engelbreth-Holm-Swarm murine sarcoma basement membrane (Sigma, Catalog No. L2020-1MG). Cultures were subsequently maintained in STEMdiff™ Forebrain Neuron Maturation Medium (STEMCELL Technologies, Catalog No. 08605) until DIV44, at which time pharmacological treatments and functional analyses were performed. Under these conditions, neuronal cultures exhibited neuronal morphology and expression of neuronal markers including MAP2 and βIII-tubulin.

### 2.3. DHA Treatment

To assess whether DHA modulates synaptic protein expression and neuronal network activity in SHINE neurons, cultures were treated with DHA (50 µM; Cayman Chemical, Ann Arbor, MI, USA, Catalog No. 90310). This concentration was selected based on a previous targeted screen in our laboratory, in which 50 µM DHA produced a robust increase in neuronal PSD-95 levels. The concentration represents an experimental in vitro exposure and should not be interpreted as directly reproducing physiological brain or circulating DHA concentrations. DHA was supplied in ethanol and diluted into neuronal maturation medium immediately before use. Vehicle-treated cultures received an equivalent concentration of ethanol, with the final solvent concentration not exceeding 0.04% (*v*/*v*) across conditions.

At DIV44, neuronal cultures were exposed to DHA or vehicle control for either 30 min or 24 h, depending on the downstream analysis. A 24 h exposure was used to assess PSD-95 abundance and neuronal network activity, whereas a 30 min exposure was used to assess acute extracellular signal-regulated kinase (ERK) and cAMP response element-binding protein (CREB) phosphorylation. Vehicle-treated cultures were exposed for the corresponding treatment duration under otherwise identical conditions.

### 2.4. Immunofluorescence Staining and Image Analysis

Neuronal cultures were fixed in 4% formaldehyde for 15 min at room temperature and washed with phosphate-buffered saline (PBS). Cells were permeabilized using 0.1% Triton X-100 (Sigma, Catalog No. X100) and blocked with Dako Protein Block Serum-Free Ready-To-Use (Fisher Scientific, Thermo Fisher Scientific, Waltham, MA, USA, Catalog No. X0909).

Cultures were incubated overnight at 4 °C with primary antibodies diluted in blocking solution, including anti-PSD-95 (4 µg/mL; Abcam, Cambridge, UK, Catalog No. ab18258) and anti-MAP2 (1:450; Invitrogen, Thermo Fisher Scientific, Carlsbad, CA, USA; Cat. No. 13-1500). After washing, Alexa Fluor-conjugated secondary antibodies were applied for 1 h at room temperature. Nuclei were counterstained with Hoechst (2.5 μg/mL, Invitrogen, Catalog No. H3570).

Images were acquired using an Opera Phenix High Content Screening System (PerkinElmer, Waltham, MA, USA). Multiple non-overlapping fields were captured per well using identical acquisition parameters across all experimental conditions.

Image analysis was performed using Columbus software version 2.9.1.699 (PerkinElmer). Nuclei were identified based on Hoechst staining, and MAP2-positive neurons were segmented using predefined fluorescence intensity thresholds. MAP2 is a neuron-enriched microtubule-associated protein predominantly localized to the soma and dendritic compartment and was used to identify neuronal processes and assess dendritic structural integrity [9]. Neurites were detected using automated neurite detection algorithms and quantified for total neurite length. PSD-95 fluorescence intensity and puncta density were measured along neuronal processes using fixed-size and intensity thresholds applied uniformly across all samples. Well-level quantitative metrics were exported for statistical analysis. Immunofluorescence analyses were performed in three independent experiments conducted on separate occasions, using one dedicated plate for each experiment. Multiple non-overlapping fields were acquired from each well and aggregated at the well level. Replicate wells within an experiment were considered technical replicates and were averaged within each experimental condition. The three independent experiments constituted the biological replicates for immunofluorescence analyses (*n* = 3).

### 2.5. Western Blot Analysis

For protein analysis, neuronal cultures were washed with ice-cold PBS and lysed on ice in radioimmunoprecipitation assay (RIPA) buffer supplemented with Halt Protease and Phosphatase Inhibitor Cocktail (100×; Thermo Scientific; Product No. 1861281) at a final concentration of 1×. Whole-cell lysates were incubated on ice for 20 min, briefly sonicated, and centrifuged at 13,000× *g* for 30 min at 4 °C to remove cellular debris. Protein concentrations were determined using a DC protein assay (Bio-Rad, Hercules, CA, USA; Cat. No. 5000111) with bovine serum albumin (BSA) standards. Equal amounts of protein were separated on 4–20% Mini-PROTEAN TGX Stain-Free gels (Bio-Rad, Cat. No. 456-8096) and transferred to polyvinylidene difluoride (PVDF) membranes. Western blot analyses were performed independently for each of the three experimental runs, with samples from the experimental conditions within a given run analyzed on the same gel. One independent gel was analyzed per experimental run, yielding three biological replicates for Western blot quantification (*n* = 3).

Membranes were blocked in 5% non-fat milk and incubated with primary antibodies against PSD-95 (4 µg/mL; Abcam, Catalog No. ab18258), β-Tubulin III (1 µg/mL, Abcam, Catalog No. ab18207), phospho-p44/42 mitogen-activated protein kinase (MAPK; ERK1/2; Thr202/Tyr204; D13.14.4E; Cell Signaling Technology, Danvers, MA, USA; Cat. No. 4370; 1:2000), total p44/42 MAPK (ERK1/2; 137F5; Cell Signaling Technology; Cat. No. 4695; 1:1000), phospho-CREB (Ser133; 87G3; Cell Signaling Technology; Cat. No. 9198; 1:1000), and total CREB (86B10; Cell Signaling Technology; Cat. No. 9104; 1:1000). Following incubation with horseradish peroxidase (HRP)-conjugated secondary antibodies, protein bands were visualized using chemiluminescent detection and imaged using a ChemiDoc imaging system (Bio-Rad).

Target protein abundance was normalized to the total protein signal from the corresponding Stain-Free lane to control for loading variability. Total-protein normalization was used rather than normalization to a single housekeeping protein and was applied consistently across experimental groups. ERK and CREB activation were expressed as the pERK/tERK and pCREB/tCREB ratios, respectively. To assess PSD-95 abundance relative to neuronal content, PSD-95 normalized volume was additionally expressed relative to β-Tubulin III normalized volume within each experimental run. PSD-95 abundance was assessed following 24 h of DHA or vehicle treatment, whereas ERK and CREB phosphorylation were assessed following 30 min of treatment. Original uncropped Western blot images are provided in Figures S2 and S3.

### 2.6. Multielectrode Array Recordings

Spontaneous neuronal network activity was recorded using a Maestro microelectrode array (MEA) system (Axion Biosystems, Atlanta, GA, USA) equipped with CytoView 48-well plates containing embedded microelectrodes (Axion Biosystems, Catalog No. M768-tMEA-48W). Human induced neurons were maintained in neuronal maturation medium until stable network activity had developed. MEA analysis was performed as a single longitudinal experiment using one 48-well MEA plate, with five replicate wells per condition.

Baseline electrophysiological recordings were obtained at DIV44 before treatment. Following baseline acquisition, individual wells were treated with DHA (50 µM) or vehicle control and returned to the incubator. The same wells were recorded again 24 h later at DIV45 using identical acquisition parameters, providing longitudinal measurements from the same neuronal cultures before and after treatment.

Recordings were performed at 37 °C in a controlled atmosphere containing 5% CO_2_. Extracellular signals were band-pass filtered between 200 Hz and 3 kHz, and action potentials were detected using an adaptive threshold set at six times the standard deviation of background noise. Each recording consisted of 20 min of spontaneous neuronal activity per well.

Electrophysiological data were analyzed using AxIS Neural Metrics software (version 4.2.1, Axion Biosystems). Mean firing rate was calculated as the total number of detected spikes divided by the 20 min recording duration and expressed in Hz. Network bursts were defined as near-synchronous spiking across multiple electrodes within a well and were identified using the default AxIS network-burst detection algorithm. Network burst frequency was calculated as the total number of network bursts divided by the recording duration and expressed in Hz, whereas average network burst duration was defined as the mean time from the first to the last spike within a network burst. The synchrony index was calculated from pairwise cross-correlations of spike trains across electrodes within each well and provides a unitless measure ranging from 0 to 1, with values closer to 1 indicating greater network synchrony.

To ensure data quality, wells were included only when at least five active electrodes were detected during the recording period. Because DIV44 and DIV45 measurements were obtained longitudinally from the same wells, measurements across these time points were treated as repeated observations.

### 2.7. Descriptive Relationship Between PSD-95 Expression and Neuronal Network Activity

To provide a descriptive visualization of the relationship between PSD-95 expression and neuronal network function across experimental conditions, group-level mean values were used. For each condition (WT, SHINE, and SHINE + DHA), mean PSD-95 abundance from the three independent Western blot experiments was plotted against the corresponding mean firing rate or synchrony index obtained from five replicate wells analyzed by MEA. Because Western blot and MEA measurements were obtained from separate experiments and were not paired at the individual-sample level, these analyses were considered descriptive only. Linear trend lines were used to visualize the direction of the condition-level relationship; no inferential correlation statistics or significance testing were applied.

### 2.8. Statistical Analysis

Statistical analyses were performed using GraphPad Prism software (version 8.4.2). Immunofluorescence and Western blot experiments were performed in three independent experimental runs (*n* = 3). For immunofluorescence analyses, technical replicate wells and imaging fields were aggregated within each independent experiment before statistical analysis. Western blot data were obtained from three independently performed experiments, with one gel analyzed per experiment. Comparisons between two groups were performed using two-tailed unpaired Student’s *t*-tests. PSD-95/β-Tubulin III ratios from corresponding WT and SHINE samples within each experimental run were compared using a two-tailed paired Student’s *t*-test. Data from these assays are presented as mean ± standard deviation (SD).

MEA data were obtained from a single 48-well plate containing five replicate wells per condition (*n* = 5). DIV44 and DIV45 measurements were obtained from the same wells and therefore constituted longitudinal repeated measurements. For the DHA treatment analysis, MEA measurements obtained from the same wells at DIV44 and DIV45 were analyzed using two-way repeated-measures ANOVA, with time (DIV44 versus DIV45) as the within-well factor and treatment (vehicle versus DHA) as the between-well factor. Sidak’s multiple comparisons test was used to compare DIV44 and DIV45 within each treatment group. Comparisons between separate experimental groups at the same time point were performed using two-tailed unpaired Student’s *t*-tests. The descriptive analyses relating group-level PSD-95 abundance to MEA parameters were not subjected to inferential correlation testing. A significance threshold of *p* < 0.05 was used for all inferential analyses. A summary of the main quantitative comparisons, including group means, variability, sample sizes, statistical tests, and exact statistical values, is provided in Table S1.

## 3. Results

### 3.1. Reduced PSD-95 Expression and Impaired Neurite Morphology in SHINE Neurons

**Figure 1:**
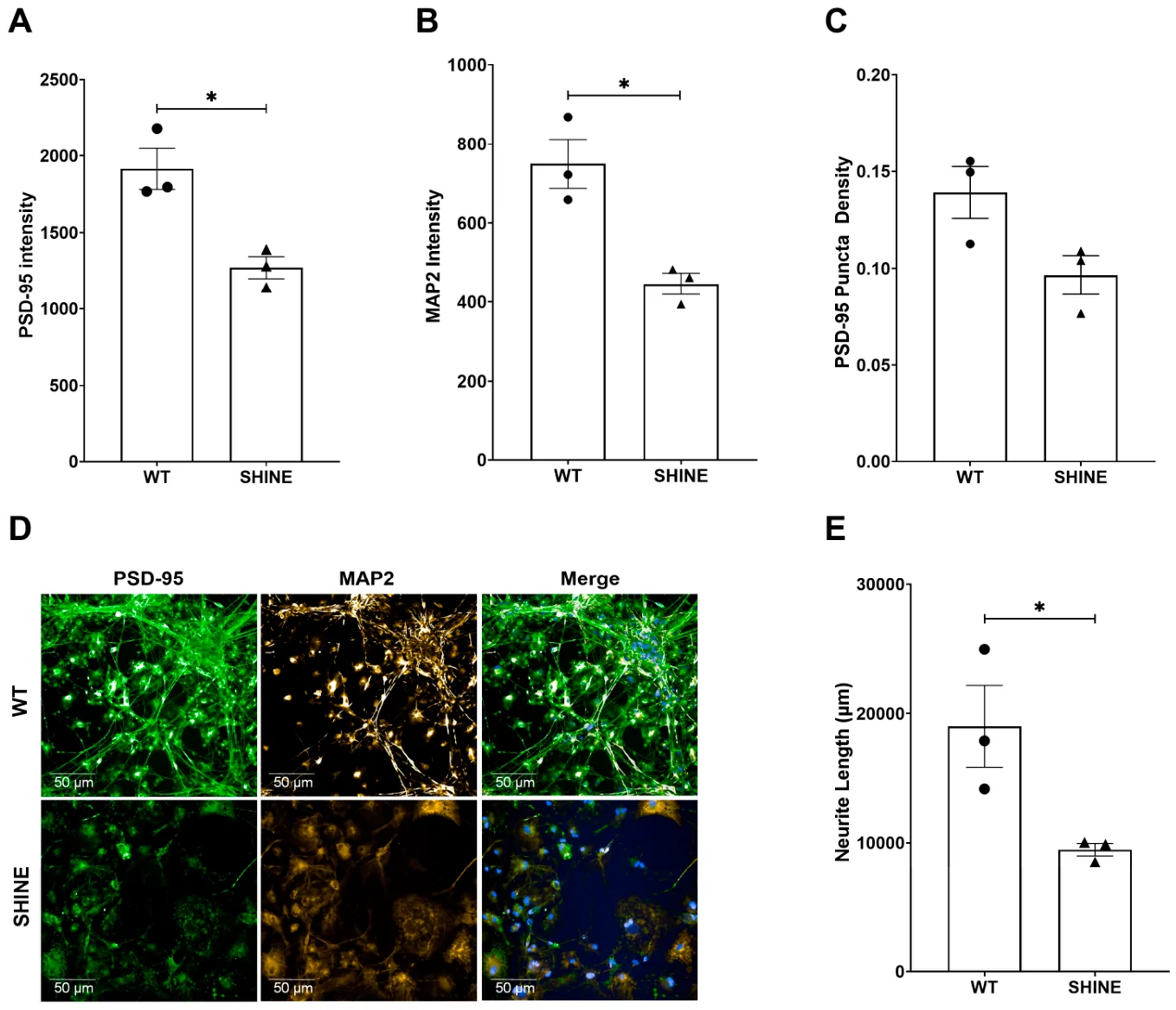
Reduced PSD-95 expression and altered neuronal morphology in SHINE iPSC-derived neurons. (**A**) PSD-95 immunofluorescence in SHINE neurons is significantly reduced compared with WT neurons. (**B**) Dendritic marker MAP2 immunofluorescence is decreased in SHINE neurons relative to WT neurons. (**C**) PSD-95 puncta density is reduced in SHINE neurons compared with WT neurons, although the difference did not reach statistical significance. (**D**) Representative immunofluorescence images of WT and SHINE neurons stained for PSD-95 (green) and MAP2 (yellow), Hoechst-positive nuclei (blue), with merged images illustrating the distribution of postsynaptic puncta along neuronal processes. Scale bars = 50 µm. (**E**) Neurite outgrowth, as reflected in total neurite length, is significantly reduced in SHINE neurons relative to WT. Data are presented as mean ± SD, with individual data points representing the three independent experiments (*n* = 3). Circles indicate vehicle-treated SHINE neurons and triangles indicate DHA-treated SHINE neurons. Statistical significance was determined using two-tailed unpaired Student’s *t*-tests. * *p* < 0.05.

**Figure 2:**
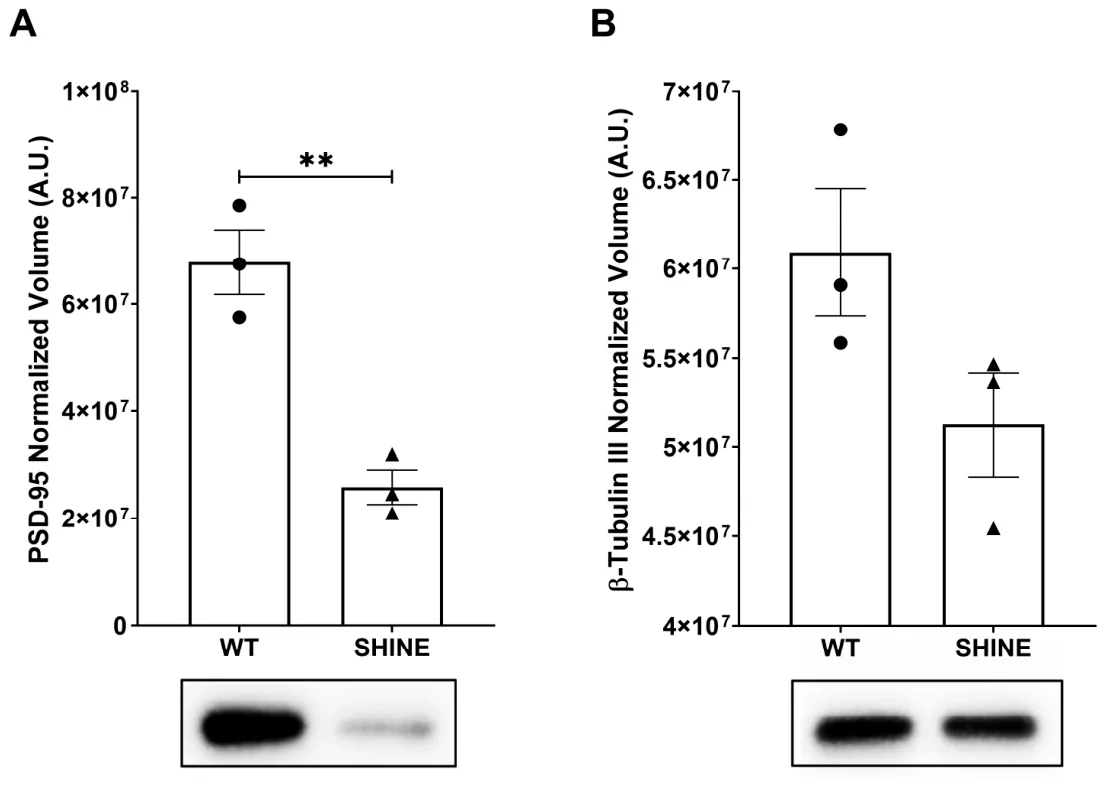
Reduced PSD-95 protein expression in SHINE neurons. Western blot quantification reveals (**A**) a significant reduction in PSD-95 normalized volume in SHINE neurons compared with WT controls and (**B**) no significant difference in β-Tubulin III levels between WT and SHINE neurons. Representative Western blot images for PSD-95 and β-Tubulin III are shown below the corresponding quantifications. Data are presented as mean ± SD, with individual points representing the three independent Western blot experiments (*n* = 3). Circles indicate vehicle-treated SHINE neurons and triangles indicate DHA-treated SHINE neurons. Statistical significance was determined using two-tailed unpaired Student’s *t*-tests. ** *p* < 0.01.

To explore the neuronal impact of DLG4 haploinsufficiency, Western blot and immunofluorescence analyses of synaptic proteins, along with assessment of neuronal morphology, were conducted on induced neurons (iNs) derived from SHINE and control individuals (Figure 1 and Figure 2).

Immunoblot quantification showed that PSD-95 protein levels in SHINE iNs were reduced to approximately 40% of those observed in control iNs, consistent with haploinsufficiency conferred by the individual’s *DLG4* (c.455delG; p.G152Afs*12) variant (Figure 2A). In contrast, β-Tubulin III, used as a neuronal control, did not differ significantly between groups (Figure 2B). When PSD-95 abundance was additionally normalized to β-Tubulin III within each experimental run, the PSD-95/β-Tubulin III ratio remained approximately 56% lower in SHINE iNs relative to WT iNs. However, this difference did not reach statistical significance (Figure S4). Immunofluorescence analysis similarly revealed an approximately 34% reduction in PSD-95 intensity in SHINE iNs compared with WT iNs (Figure 1A). In addition, MAP2 immunofluorescence was approximately 40% reduced in SHINE iNs (Figure 1B), indicating reduced dendrite-associated MAP2 signal. PSD-95 puncta density, defined as the number of PSD-95 puncta detected along neuronal processes, was also reduced in SHINE iNs by approximately 30%, although this difference did not reach statistical significance (Figure 1C). Representative images illustrate reduced PSD-95 signal together with altered neurite architecture in SHINE iNs (Figure 1D). In keeping with these findings, total neurite length was reduced by approximately 50% in SHINE iNs compared with WT iNs (Figure 1E); the lower MAP2 signal was consistent with diminished neuronal process development and altered structural maturation.

Together, these results demonstrate that SHINE neurons exhibit reduced PSD-95 expression and impaired neurite morphology, consistent with disrupted postsynaptic organization associated with DLG4 haploinsufficiency.

### 3.2. SHINE Neurons Exhibit Impaired Neuronal Network Activity

**Figure 3:**
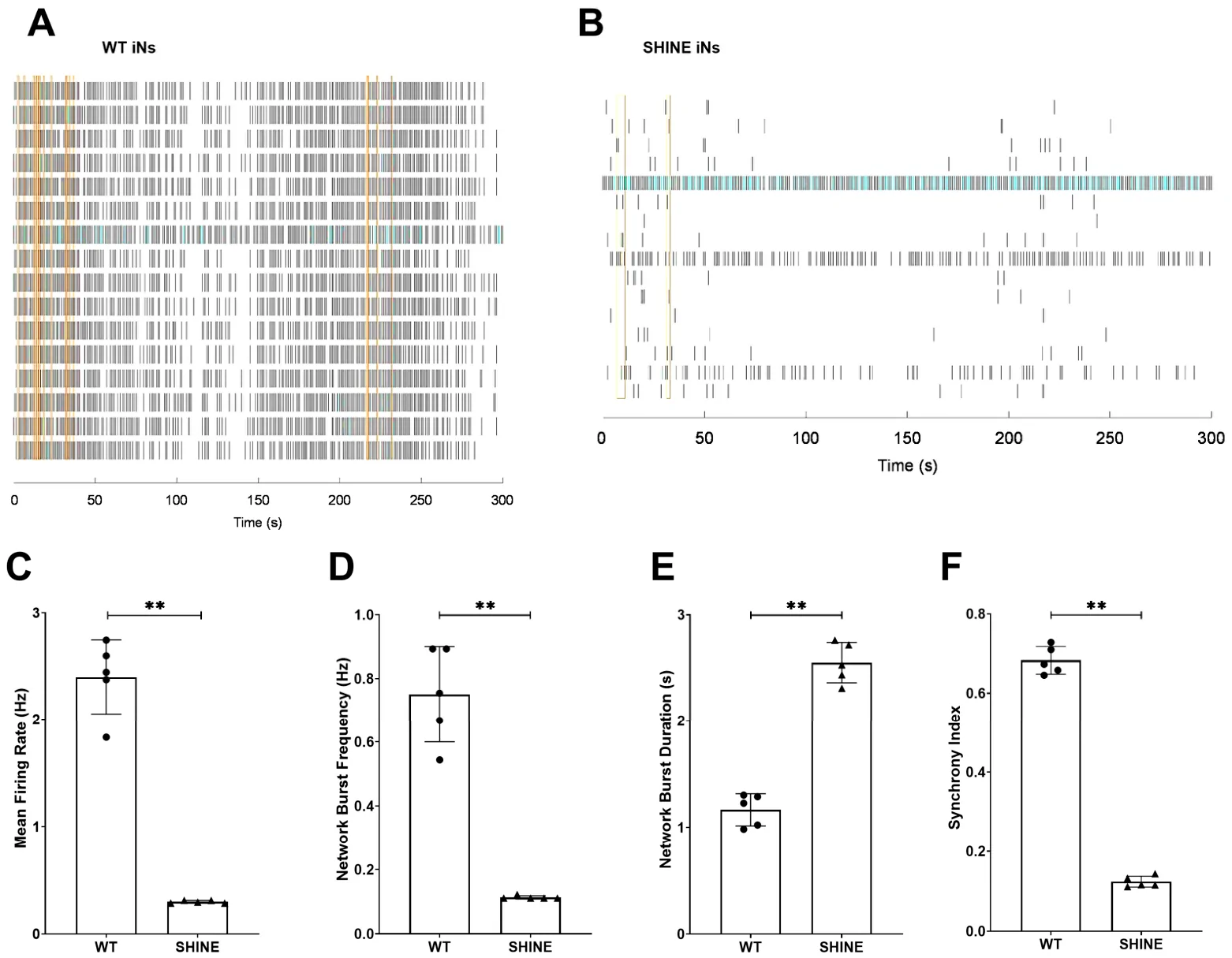
Impaired neuronal network activity in SHINE-induced neurons. (**A**,**B**) Representative raster plots showing spontaneous neuronal activity recorded using a multielectrode array (MEA) from wild-type (WT) induced neurons (iNs) (**A**) and SHINE iNs (**B**) over a 300 s recording period. WT neurons display frequent and coordinated firing patterns, whereas SHINE neurons exhibit markedly reduced and less synchronized activity. (**C**) Quantification of mean firing rate demonstrating significantly reduced neuronal firing in SHINE neurons compared with WT controls. (**D**) Network burst frequency analysis showing decreased burst occurrence in SHINE neurons. (**E**) Average network burst duration indicating prolonged burst events in SHINE neurons relative to WT. (**F**) Synchrony index analysis demonstrating reduced network synchrony in SHINE neurons compared with WT. Data are presented as mean ± SD, with individual points representing replicate wells from a single MEA experiment (five wells per group). Circles indicate vehicle-treated SHINE neurons and triangles indicate DHA-treated SHINE neurons. In the raster plots, gray tick marks indicate detected spikes, blue tick marks indicate spikes occurring within bursts, and yellow outlined regions indicate detected network bursts. Statistical significance was determined using two-tailed unpaired Student’s *t*-tests. ** *p* < 0.01.

To determine whether reduced PSD-95 expression in SHINE iNs was associated with altered neuronal network activity, MEA recordings were conducted in WT and SHINE iNs at DIV45 (Figure 3). A marked reduction in mean firing rate was observed in SHINE iNs compared with WT iNs, corresponding to an approximately 8-fold decrease (Figure 3C). Consistent with reduced neuronal excitability, network burst frequency was also significantly decreased in SHINE iNs, with an approximately 6.6-fold reduction relative to WT iNs (Figure 3D). In addition to reduced firing and bursting rates, SHINE neuronal networks displayed altered burst dynamics. Network burst duration was increased by approximately 2.2-fold in SHINE iNs compared with WT iNs (Figure 3E), indicating prolonged but less frequent network events.

Finally, neuronal network synchrony, captured by the synchrony index, was markedly reduced in SHINE iNs compared with WT controls, corresponding to an approximately 5.5-fold decrease (Figure 3F), demonstrating impaired coordinated neuronal activity across the network. Together, these findings show impaired neuronal network activity in SHINE iNs, characterized by reduced firing rates, decreased burst frequency, prolonged burst duration, and diminished network synchrony.

### 3.3. DHA Increases PSD-95 Expression and Improves Neuronal Network Activity

DHA, in addition to its known role as a critical neuronal membrane component, has also been identified as a robust inducer of neuronal PSD-95 levels in a targeted screen previously conducted in our laboratory (Figure S1). To determine whether DHA treatment could rescue synaptic deficits in SHINE neurons, analyses of SHINE iNs treated with DHA (50 μM) for 24 h were undertaken.

**Figure 4:**
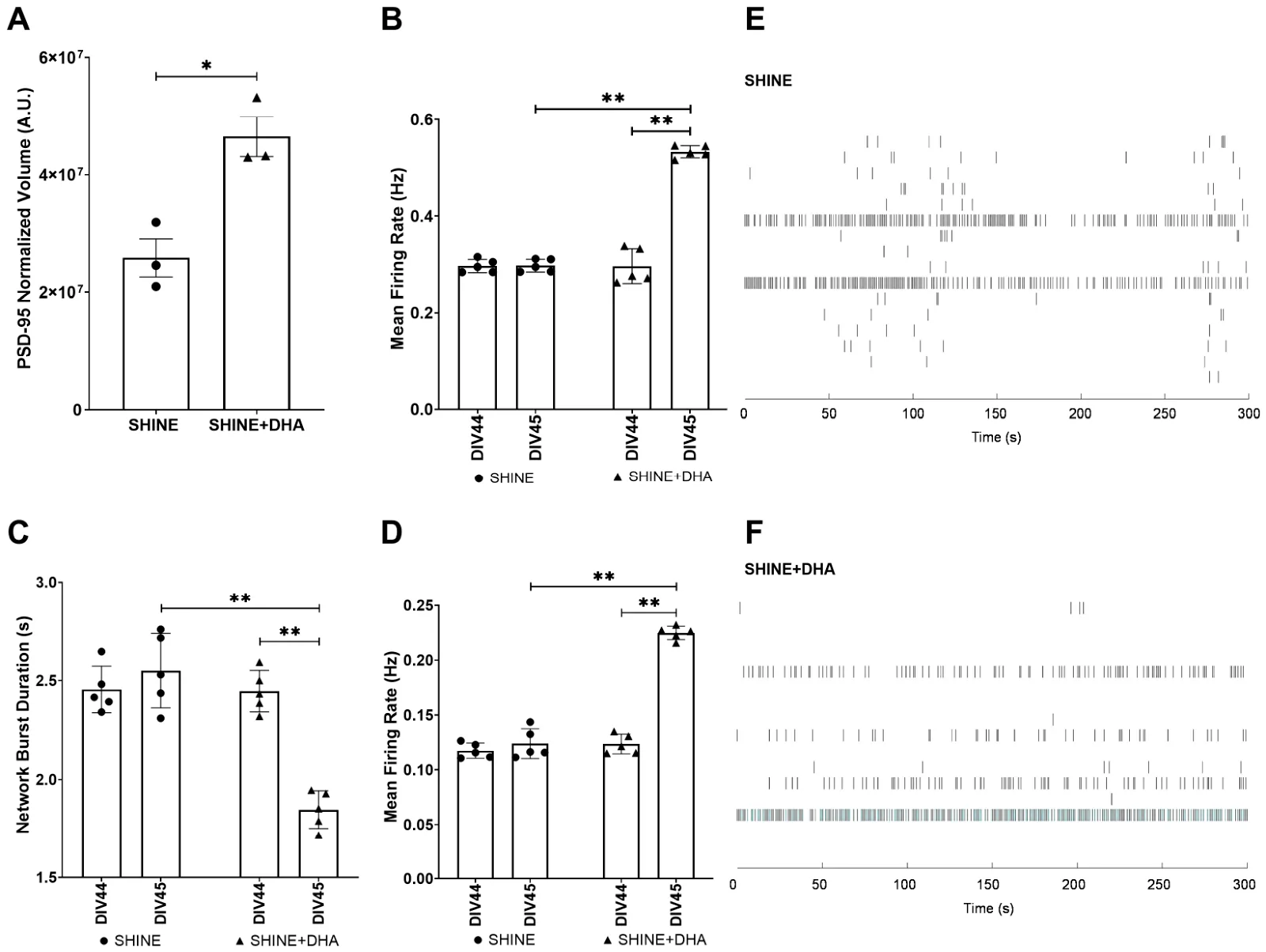
DHA treatment increases PSD-95 expression and improves neuronal network activity in SHINE neurons. (**A**) Western blot quantification showed increased PSD-95 protein levels in DHA-treated SHINE neurons relative to vehicle-treated SHINE neurons. (**B**–**D**) MEA measurements at DIV44 before treatment and DIV45 following treatment showing (**B**) mean firing rate, (**C**) average network burst duration, and (**D**) synchrony index in vehicle-treated and DHA-treated SHINE neurons. DHA-treated SHINE neurons showed increased firing rate, reduced burst duration, and increased synchrony, whereas vehicle-treated SHINE neurons remained stable across time. (**E**,**F**) Representative raster plots of spontaneous neuronal activity recorded by MEA from vehicle-treated SHINE neurons (**E**) and DHA-treated SHINE neurons (**F**) over a 300 s recording period. DHA-treated SHINE neurons displayed increased spiking activity and altered network firing patterns. Blue-highlighted segments indicate detected network burst events. Data are presented as mean ± SD, with individual points representing replicate wells from a single MEA experiment (five wells per treatment condition). Circles indicate vehicle-treated SHINE neurons and triangles indicate DHA-treated SHINE neurons. In the raster plots, gray tick marks indicate detected spikes, and blue tick marks indicate spikes occurring within bursts. PSD-95 comparisons were performed using a two-tailed unpaired Student’s *t*-test. MEA data were analyzed using two-way repeated-measures ANOVA followed by Sidak’s multiple comparisons test. * *p* < 0.05, ** *p* < 0.01.

In keeping with our earlier results, Western blot analysis revealed an almost doubling of PSD-95 protein levels in SHINE iNs following DHA treatment (Figure 4A). To assess whether this increase in PSD-95 was associated with functional improvements in neuronal network activity, MEA recordings were performed. DHA treatment significantly increased mean firing rate by approximately 1.8-fold compared with vehicle SHINE iNs (Figure 4B) and significantly reduced network burst duration by approximately 28% (Figure 4C), indicating partial normalization of burst dynamics. Consistent with these findings, neuronal network synchrony was significantly increased by approximately 1.8-fold following DHA treatment (Figure 4D). Longitudinal analysis of the same wells from DIV44 to DIV45 further showed significant time-by-treatment interactions for mean firing rate (*p* < 0.01), network burst duration (*p* = 0.01), and synchrony index (*p* < 0.01). Vehicle-treated SHINE iNs remained stable across time, whereas DHA-treated SHINE iNs showed increased firing rate, reduced burst duration, and increased synchrony. Representative raster plots illustrate increased neuronal firing and improved network organization in DHA-treated SHINE iNs compared with vehicle SHINE controls (Figure 4E,F).

To determine whether the DHA response was restricted to SHINE neurons, WT and SHINE iNs were treated in parallel with vehicle or DHA and analyzed by immunofluorescence and MEA (Figure S5). In WT iNs, DHA significantly increased PSD-95 immunofluorescence intensity and PSD-95 puncta density. In the corresponding SHINE immunofluorescence comparisons, these increases did not reach statistical significance, despite the increase in total PSD-95 detected by Western blot (Figure 4A). MAP2 intensity showed an overall treatment effect, with no significant genotype-by-treatment interaction. Analysis of DIV44-to-DIV45 changes in MEA activity revealed significant genotype-by-treatment interactions for mean firing rate, network burst duration, and synchrony index. DHA-associated changes in these measures were significant in SHINE iNs but not in WT iNs. Together, these findings indicate that the effects of DHA on PSD-95 are not restricted to SHINE neurons, whereas the network-level response differed between WT and SHINE iNs.

To visualize whether changes in PSD-95 abundance paralleled changes in neuronal network activity across experimental conditions, group-level mean PSD-95 values were plotted against the corresponding mean firing rate and synchrony index for WT, SHINE, and SHINE+DHA conditions (Figure S6). Conditions with higher mean PSD-95 abundance also showed higher mean firing rate and network synchrony. Because Western blot and MEA measurements were obtained from separate experiments and were not paired at the individual-sample level, these analyses are descriptive and do not establish a statistically significant sample-level correlation. Nevertheless, the condition-level pattern illustrates that changes in PSD-95 abundance occur in the same direction as changes in neuronal network function. Despite these improvements, WT iNs generally exhibited higher firing and synchrony than DHA-treated SHINE iNs, suggesting that pharmacologic restoration of PSD-95 does not fully recapitulate the functional properties associated with endogenous PSD-95 expression.

Together, these results demonstrate that DHA increases PSD-95 abundance and improves neuronal network activity in SHINE neurons. In contrast, comparison with WT neurons indicates that the molecular and network responses to DHA differ between the two neuronal cultures.

### 3.4. DHA Is Associated with Trends Toward Increased ERK and CREB Phosphorylation

**Figure 5:**
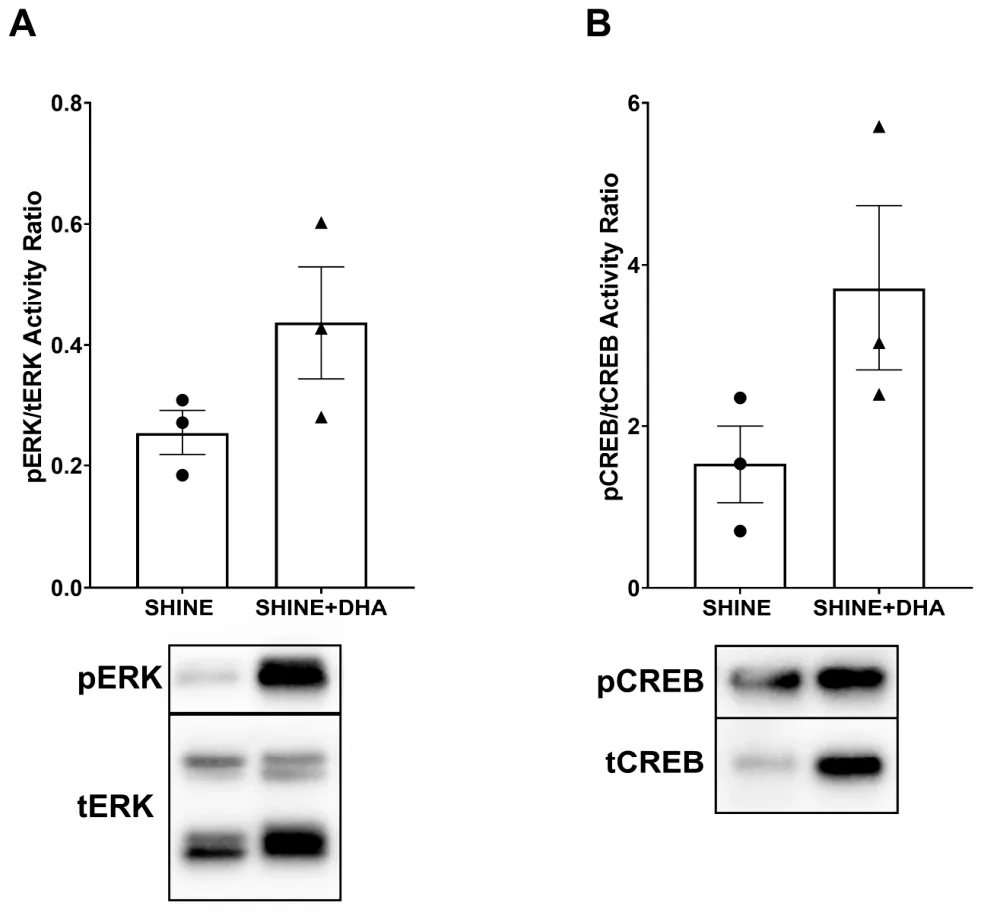
DHA treatment is associated with trends toward increased ERK and CREB phosphorylation in SHINE neurons. (**A**) ERK activation as measured by the phosphorylated ERK (pERK) to total ERK (tERK) ratio in SHINE neurons following 30 min of vehicle control (SHINE) or DHA (SHINE + DHA). DHA treatment increased the pERK/tERK ratio compared with SHINE controls, although the difference did not reach statistical significance. Representative Western blot images for pERK/tERK are shown below the corresponding quantification. (**B**) Quantification of CREB activation measured as the phosphorylated CREB (pCREB) to total CREB (tCREB) ratio in SHINE neurons treated with vehicle control or DHA. DHA-treated neurons exhibited higher pCREB/tCREB ratios compared with SHINE controls. Representative Western blot images for pCREB/tCREB are shown below the corresponding quantification. Data are presented as mean ± SD, with individual points representing the three independent Western blot experiments (*n* = 3). Circles indicate vehicle-treated SHINE neurons, and triangles indicate DHA-treated SHINE neurons. Statistical significance was determined using two-tailed unpaired Student’s *t*-tests.

To investigate potential intracellular signaling pathways associated with the DHA-mediated increase in PSD-95 levels, we assessed ERK and CREB phosphorylation in SHINE iNs following 30 min DHA or vehicle treatment (Figure 5).

Western blot analysis revealed an approximately 1.7-fold increase in the pERK/tERK ratio in DHA-treated SHINE iNs compared with vehicle SHINE controls (Figure 5A). Although this increase did not reach statistical significance, a trend toward increased ERK phosphorylation was observed following DHA treatment. Similarly, DHA treatment increased the pCREB/tCREB ratio by approximately 2.4-fold compared with SHINE control iNs (Figure 5B). While this difference was also not statistically significant, a similar trend toward increased CREB phosphorylation was observed. Together, these results indicate trends toward increased ERK and CREB phosphorylation following DHA treatment in SHINE iNs, although neither effect reached statistical significance.

## 4. Discussion

### 4.1. Reduced PSD-95 Abundance and Altered Neuronal Morphology in SHINE Neurons

Synaptic scaffold proteins are central organizers of excitatory synapses, and disruption of these proteins has increasingly been recognized as an important mechanism underlying neurodevelopmental disorders [5,11]. SHINE syndrome provides a particularly informative model because pathogenic variants in *DLG4* reduce the abundance of PSD-95, a major postsynaptic scaffold [10,11]. In the present study, SHINE iNs showed reduced PSD-95 abundance compared with WT iNs, consistent with DLG4 haploinsufficiency.

In addition to lower PSD-95 immunofluorescence intensity and reduced PSD-95 puncta density, decreased MAP2 signal and shorter neurites were also observed, indicating alterations in both postsynaptic organization and neuronal architecture in SHINE iNs. MAP2 is a neuron-enriched microtubule-associated protein that contributes to the organization and stabilization of the dendritic cytoskeleton and is commonly used to visualize neuronal somata and dendritic processes [9]. The reduced MAP2 immunofluorescence observed in SHINE neurons, together with the marked reduction in total neurite length, therefore supports an alteration in neuronal structural development. Because the present analysis measured overall MAP2 fluorescence rather than MAP2 abundance normalized to individual dendritic area, the reduction cannot distinguish between lower MAP2 expression per neuron and a decrease in the amount or complexity of MAP2-positive neuronal processes. Nevertheless, the reduction in both MAP2 signal and neurite length suggests that DLG4 haploinsufficiency is associated not only with reduced postsynaptic scaffold abundance but also with broader abnormalities in neuronal morphology. These changes may reflect impaired neuronal maturation, reduced neurite stability, or a combination of both.

These structural changes, alongside a major reduction in PSD-95, align with its known role as a central scaffold, stabilizing, among other proteins, AMPA receptors, as well as supporting activity-dependent synapse maturation [2,21]. Reduced PSD-95 dosage would therefore be expected to weaken the postsynaptic architecture and activity-dependent reinforcement required to stabilize developing neuronal processes. In this regard, SHINE syndrome is thought to involve impaired postsynaptic signaling and disrupted excitatory synaptic function [5]. These structural abnormalities likely underpin the network dysfunction described in the next section.

### 4.2. DLG4 Haploinsufficiency Is Associated with Impaired Neuronal Network Activity

The molecular and structural abnormalities observed in SHINE iNs were accompanied by a clear functional impairment at the network level as measured by MEA. SHINE iNs showed reduced firing rate, decreased burst frequency, prolonged burst duration, and reduced synchrony when compared with WT iNs. Consistent with these findings, a recent study using the same CH1371 frameshift line, together with additional *DLG4*-mutant iPSC-derived neuronal lines, identified genotype-dependent electrophysiological abnormalities at both single-neuron and network levels using high-density MEA analysis. Importantly, restoration of *DLG4* expression increased PSD-95 abundance and shifted electrophysiological phenotypes toward the WT state [22].

In cultured neuronal networks, firing, bursting, and synchrony reflect the formation of stable synaptic connections and coordinated recruitment of neurons into network events [23,24]. The combination of reduced firing, weaker bursting, and lower synchrony points to impaired excitatory network organization rather than an isolated change in neuronal excitability. Reduced firing rate and burst frequency indicate weaker network recruitment, whereas reduced synchrony indicates impaired coordination across the network. The prolonged burst duration further suggests that when SHINE neuronal networks do generate burst events, those events are less efficiently organized. The pattern therefore reflects a coordinated network-level impairment rather than an isolated change in a single electrophysiological parameter. The contribution of AMPA receptor-mediated transmission to the spontaneous network activity was not directly assessed using pharmacological antagonists such as CNQX; therefore, the present MEA findings reflect spontaneous neuronal network activity and cannot be attributed specifically to AMPA receptor activation.

This functional impairment is consistent with the known role of PSD-95 in excitatory synapse stabilization. PSD-95 regulates AMPA receptor stabilization, postsynaptic organization, and activity-dependent synaptic maturation, processes required for efficient communication between excitatory neurons [2,6,7,21].

Loss of PSD-95 would therefore be expected to weaken the synaptic architecture needed for coordinated firing and synchronized bursting. The MEA data provide the functional counterpart to the molecular deficit, showing that PSD-95 reduction is accompanied by impaired network-level behavior.

The reduction in synchrony is particularly important because synchronized bursting reflects coordinated communication across neuronal populations, not simply activity within individual cells. Similar synchrony deficits have been reported in human neuronal models of SHANK3-related disorders and Fragile X syndrome (FXS), caused by silencing of the fragile X messenger ribonucleoprotein 1 (*FMR1*) gene, supporting the broader view that synaptic gene disruption can propagate to network-level dysfunction [25,26]. The present findings identify a similar network-level disturbance in SHINE iNs. Together with the structural findings, these data support a model in which reduced PSD-95 weakens excitatory synaptic connectivity, yielding a network that fires less, bursts less efficiently, and synchronizes poorly.

### 4.3. DHA Partially Restores PSD-95 Expression and Improves Neuronal Network Activity in SHINE Neurons

Having confirmed that SHINE iNs show the expected reduction in PSD-95 levels as well as impaired network organization, we next asked whether these disease-relevant features were modifiable. In this regard, DHA, the major long-chain ω-3 polyunsaturated fatty acid (PUFA), is highly enriched in neuronal membranes, where its highly unsaturated acyl chain increases membrane fluidity and promotes membrane curvature [15,16]. These biophysical properties influence phospholipid organization, membrane-dependent signaling, synaptic membrane architecture, and postsynaptic scaffold regulation [16,17]. Numerous studies have demonstrated that DHA increases PSD-95 abundance as well as other synaptic proteins, associated with enhanced synaptogenesis and neurite growth in various neuronal systems [18,19,20,27]. Our previous work has confirmed this DHA-mediated PSD-95 increase, showing that DHA, more than the other major long-chain ω-3 fatty acid, eicosapentaenoic acid (EPA; 20:5n-3) formulations or EPA-dominant DHA:EPA compositions, preferentially increased PSD-95 abundance, PSD-95 puncta density, PSD-95-synaptophysin-1 colocalization, and glutamatergic signaling markers in differentiated neuronal cells [28].

We show here that DHA treatment shifted SHINE iNs toward a more organized molecular and functional state, increasing PSD-95 protein levels, neuronal firing, and network synchrony while reducing burst duration. At the group level, conditions with higher mean PSD-95 abundance also exhibited higher mean firing rate and synchrony. Importantly, although DHA improved several features disrupted by DLG4 haploinsufficiency, treated SHINE iNs did not fully reach the WT pattern of activity.

The relationship between increased PSD-95 abundance and improved network activity remains difficult to disentangle. Although DHA increased both PSD-95 expression and network organization, the present study does not establish whether the functional improvement is mediated primarily through restoration of PSD-95 or through additional effects of DHA on neuronal membrane biology. Nevertheless, the parallel condition-level changes in PSD-95 abundance, firing rate, and synchrony are consistent with an association between restoration of postsynaptic scaffold integrity and improved network coordination in SHINE iNs.

Finally, because DHA has been linked to activity-dependent plasticity pathways, we examined ERK/CREB signaling as a potential contributor to the DHA response. DHA-treated SHINE iNs showed trends toward higher pERK/tERK and pCREB/tCREB ratios, although neither comparison reached statistical significance. These findings should therefore be viewed as exploratory and do not establish ERK/CREB signaling as a mechanistic mediator of the DHA response.

Overall, DHA improved the two central disease-relevant abnormalities identified in this model, reduced PSD-95 abundance and impaired network organization, while not fully restoring the WT state. These data support further investigation of DHA and related lipid-based approaches as strategies to modify synaptic and network dysfunction in DLG4-related synaptopathy.

### 4.4. Limitations and Future Directions

A limitation of the present study is the use of a single patient-derived SHINE line and a single unrelated WT control. Because the lines differ in their genetic backgrounds, the observed differences cannot be attributed specifically to DLG4 haploinsufficiency. Although β-III tubulin abundance did not differ significantly between groups, differences in differentiation efficiency, neuronal maturity, or other line-specific properties cannot be excluded. Confirmation in additional patient lines or an isogenic model will be required to establish genotype-specific effects. The MEA findings were obtained from a single neuronal differentiation batch and one MEA plate. Although multiple wells were analyzed within this experiment, replication in an independent differentiation and MEA plate will be important to confirm the network phenotypes observed here.

The findings were obtained using an in vitro model of human induced neurons, which does not fully recapitulate the structural organization and connectivity of neuronal circuits in the developing brain. Although this system allows controlled investigation of synaptic and electrophysiological phenotypes, additional studies using more complex experimental models, such as organoid or in vivo systems, will be required to determine whether the observed effects extend to intact neural circuits.

Second, the present study focused primarily on excitatory neuronal populations. In the brain, neuronal network activity emerges from interactions between excitatory neurons, inhibitory interneurons, and glial cells. Future studies incorporating co-culture systems that include additional brain cell types, such as inhibitory interneurons or astrocytes, may provide further insight into how DLG4 haploinsufficiency affects circuit-level organization and whether DHA influences these cellular interactions.

Finally, while DHA improved PSD-95 levels and neuronal network activity in SHINE neurons, the mechanisms underlying these effects remain incompletely defined. The ERK and CREB findings were exploratory and did not establish these pathways as mediators of the DHA response. Future time-course and pathway inhibition studies in SHINE iNs will be required to determine whether phosphoinositide 3-kinase (PI3K)/protein kinase B (Akt)-ribosomal protein S6 (S6), ERK/CREB, or other lipid-sensitive mechanisms drive DHA-associated changes in PSD-95 and network activity.

Together, these considerations highlight the need for further investigation in more complex experimental systems to better define the mechanisms and therapeutic potential of DHA in DLG4-related synaptopathy.

In summary, this study establishes SHINE iNs as a disease-relevant human neuronal model in which molecular, structural, and functional abnormalities were observed relative to an unrelated WT control. The main findings are that a functional and structural phenotype has been delineated, and these disease-relevant abnormalities are modifiable. DHA increased PSD-95 abundance and improved neuronal network activity in SHINE iNs, indicating that PSD-95 abundance and network organization can be shifted in a favorable direction in a human model of SHINE syndrome. These findings identify DHA as a candidate synaptic modifier and support further evaluation of lipid-based therapeutic strategies for SHINE syndrome.

## Data Availability

The raw data supporting the conclusions of this article will be made available by the authors, without undue reservation.

## Supplementary Materials

The following supporting information can be downloaded at: https://www.mdpi.com/article/10.3390/cells15181688/s1, Table S1. Summary of quantitative protein, structural, and neuronal network measurements. Figure S1. Preliminary targeted screen of selected candidate compounds for effects on PSD-95 in differentiated SH-SY5Y cells. Differentiated SH-SY5Y cells were treated for 24 h with vehicle or the five highest-ranking candidate compounds from an initial 18-compound screen: ketamine (1 µM), DHA (50 µM), allopurinol (35 µM), valsartan (5 µM), and fluoxetine (5 µM). PSD-95 immunofluorescence intensity was used as the screening readout. The figure shows the five highest-ranking candidates based on the magnitude of change relative to vehicle-treated controls. DHA produced the largest increase in PSD-95 immunofluorescence intensity. Data are presented as mean ± SD, with *n* = 3 independent experiments. Statistical analysis was performed using one-way ANOVA followed by Dunnett’s multiple-comparisons test. ** *p* < 0.01 vs. vehicle. Figure S2. Uncropped Western blot images: WT versus SHINE neurons. (**A**) Stain-Free total-protein image corresponding to the WT and SHINE samples analyzed by Western blotting. (**B**) Uncropped PSD-95 blot (95 kDa). (**C**) Uncropped βIII-tubulin blot (55 kDa). Three independent biological replicates are shown for each condition (*n* = 3). All samples used for each comparison were run on the same gel. Lanes not included in the representative cropped images presented in the manuscript are indicated with an “X”. No lanes were rearranged or spliced. Figure S3. Uncropped Western blot images: SHINE neurons treated with DHA. (**A**) Stain-Free total-protein image corresponding to SHINE vehicle-control and SHINE+DHA samples following 24 h treatment. (**B**) Stain-Free total-protein image corresponding to SHINE vehicle-control and SHINE+DHA samples following 30 min treatment. (**C**) Uncropped PSD-95 blot (95 kDa) following 24 h treatment. (**D**) Uncropped phospho-p44/42 MAPK (pERK1/2; Thr202/Tyr204; 44/42 kDa) blot following 30 min treatment. (**E**) Uncropped total p44/42 MAPK (tERK1/2; 44/42 kDa) blot corresponding to (**D**). (**F**) Uncropped phospho-CREB (Ser133; 43 kDa) blot following 30 min treatment. (**G**) Uncropped total CREB (43 kDa) blot corresponding to (**F**). Three independent biological replicates are shown for each condition (*n* = 3). All samples used for each comparison were run on the same gel. Lanes not included in the representative cropped images presented in the manuscript are indicated with an “X”. No lanes were rearranged or spliced. Figure S4. PSD-95 abundance normalized to β-tubulin III in WT and SHINE iPSC-derived neurons. PSD-95 abundance was normalized to β-Tubulin III within each experimental run to assess PSD-95 abundance relative to neuronal content. The PSD-95/β-Tubulin III ratio was lower in SHINE neurons relative to WT neurons, although the difference did not reach statistical significance. Data are presented as mean ± SD, with individual data points representing the three independent experiments (*n* = 3). Statistical significance was determined using a two-tailed paired Student’s *t*-test. Figure S5. Effects of DHA treatment on synaptic markers and neuronal network activity in WT and SHINE neurons. (**A**) PSD-95 immunofluorescence intensity is increased following DHA treatment in WT neurons, whereas the increase in SHINE neurons is not significant. (**B**) PSD-95 puncta density is increased following DHA treatment in WT neurons, with no significant change in SHINE neurons. (**C**) MAP2 immunofluorescence intensity in WT and SHINE neurons following vehicle or DHA treatment. (**D**–**F**) Changes from DIV44 to DIV45 (Δ = DIV45 − DIV44) in (**D**) mean firing rate, (**E**) network burst duration, and (**F**) synchrony index. DHA treatment significantly increased mean firing rate and synchrony and reduced network burst duration in SHINE neurons, whereas no significant treatment-related differences were observed in WT neurons. Data are presented as mean ± SD. Individual data points represent three independent experiments in (**A**–**C**) and replicate wells from a single MEA experiment in (**D**–**F**) (*n* = 5 wells per condition). Data in (**A**–**C**) were analyzed using two-way repeated-measures ANOVA with experimental run as the matched factor, and data in (**D**–**F**) using ordinary two-way ANOVA of change scores, followed by Sidak’s multiple comparisons test. * *p* < 0.05, ** *p* < 0.01. Figure S6. Descriptive relationship between PSD-95 abundance and neuronal network activity across experimental conditions. (**A**) Mean PSD-95 abundance for each experimental condition (WT, SHINE, and SHINE + DHA) plotted against the corresponding mean firing rate. (**B**) Mean PSD-95 abundance plotted against the corresponding mean synchrony index. Each point represents one experimental condition and combines the mean PSD-95 value obtained from three independent Western blot experiments with the corresponding mean MEA value obtained from five neuronal clones. Linear trend lines are shown for descriptive visualization only. Because Western blot and MEA measurements were obtained from separate experiments and were not paired at the individual-sample level, no inferential correlation statistics were performed.

## Abbreviations

The following abbreviations are used in this manuscript:

ADHD	Attention-deficit/hyperactivity disorder
Akt	Protein kinase B
AMPA	α-Amino-3-hydroxy-5-methyl-4-isoxazolepropionic acid
ASD	Autism spectrum disorder
BSA	Bovine serum albumin
CREB	cAMP response element-binding protein
DHA	Docosahexaenoic acid
DIV	Day in vitro
DLG4	Discs large MAGUK scaffold protein 4
EPA	Eicosapentaenoic acid
ERK	Extracellular signal-regulated kinase
FMR1	Fragile X Messenger Ribonucleoprotein 1
FXS	Fragile X syndrome
HRP	Horseradish peroxidase
iPSC	Induced pluripotent stem cell
iNs	Induced neurons
MAP2	Microtubule-associated protein 2
MAPK	Mitogen-activated protein kinase
MEA	Microelectrode array
NMD	Nonsense-mediated decay
NMDA	N-Methyl-D-aspartate
NPC	Neural progenitor cell
PBS	Phosphate-buffered saline
PI3K	Phosphoinositide 3-kinase
PLO	Poly-L-ornithine
PSD	Postsynaptic density
PSD-95	Postsynaptic density protein 95
PUFA	Polyunsaturated fatty acid
PVDF	Polyvinylidene difluoride
RIPA	Radioimmunoprecipitation assay
S6	Ribosomal protein S6
SD	Standard deviation
SHINE	Sleep disturbances, Hypotonia, Intellectual disability, Neurological disorders, and Epilepsy
WT	Wild type
