# Supplementary material for: Human Neuronal Modeling of DLG4-Related Synaptopathy Reveals Network Dysfunction and Partial Rescue by Omega-3 Fatty Acid Docosahexaenoic Acid (DHA)
Source: Cells. 2026 Sep 17;15(18):1688. doi: 10.3390/cells15181688 (PMC13605776; doi:10.3390/cells15181688)
Supplement: Supplementary file 1 [file cells-15-01688-s001.zip › cells-4558921-supplementary.pdf]

Table S1. Summary of quantitative protein, structural, and neuronal network measurements

Measurement	Group means	n	Statistical analysis	Effect estimate / 95% CI	Test statistic	Exact *p* value
**Baseline molecular and structural measurements**						
PSD-95 immunofluorescence intensity	WT: 1915; SHINE: 1271	3 experiments/group	Two-tailed unpaired Student's t-test	SHINE − WT: −643.9; 95% CI −1065 to −222.3	t(4) = 4.240	0.0133
MAP2 immunofluorescence intensity	WT: 749.6; SHINE: 446.5	3 experiments/group	Two-tailed unpaired Student's t-test	SHINE − WT: −303.1; 95% CI −491.2 to −115.1	t(4) = 4.476	0.0110
PSD-95 puncta density	WT: 0.1391; SHINE: 0.09654	3 experiments/group	Two-tailed unpaired Student's t-test	SHINE − WT: −0.04256; 95% CI −0.08899 to 0.003862	t(4) = 2.546	0.0636
Total neurite length (μm)	WT: 19,011; SHINE: 9467	3 experiments/group	Two-tailed unpaired Student's t-test	SHINE − WT: −9544; 95% CI −18,442 to −646.4	t(4) = 2.978	0.0408
PSD-95 normalized volume	WT: 67,846,076; SHINE: 25,807,946	3 experiments/group	Two-tailed unpaired Student's t-test	SHINE − WT: −42,038,129; 95% CI −61,151,546 to −22,924,713	t(4) = 6.107	0.0036
β-tubulin III normalized volume	WT: 60,918,911; SHINE: 51,249,658	3 experiments/group	Two-tailed unpaired Student's t-test	SHINE − WT: −9,669,252; 95% CI −22,499,476 to 3,160,971	t(4) = 2.092	0.1045
PSD-95/β-tubulin III ratio	WT: 1.132; SHINE: 0.502	3 paired experiments	Two-tailed paired Student's t-test	SHINE − WT: −0.6305; 95% CI −1.332 to 0.07142	t(2) = 3.865	0.0609
**Baseline neuronal network measurements at DIV45**						
Mean firing rate (Hz)	WT: 2.398; SHINE: 0.2966	5 wells/group	Two-tailed unpaired Student's t-test	SHINE − WT: −2.102; 95% CI −2.460 to −1.744	t(8) = 13.53	<0.01
Network burst frequency	WT: 0.7502; SHINE: 0.1132	5 wells/group	Two-tailed unpaired Student's t-test	SHINE − WT: −0.6370; 95% CI −0.7918 to −0.4821	t(8) = 9.486	<0.01
Network burst duration (s)	WT: 1.164; SHINE: 2.550	5 wells/group	Two-tailed unpaired Student's t-test	SHINE − WT: 1.386; 95% CI 1.137 to 1.635	t(8) = 12.83	<0.01
Synchrony index	WT: 0.6828; SHINE: 0.1239	5 wells/group	Two-tailed unpaired Student's t-test	SHINE − WT: −0.5589; 95% CI −0.5975 to −0.5203	t(8) = 33.40	<0.01
**DHA response in SHINE neurons**						
PSD-95 normalized volume	Vehicle: 25,807,946; DHA: 46,501,246	3 experiments/group	Two-tailed unpaired Student's t-test	DHA − Vehicle: 20,693,299; 95% CI 7,711,726 to 33,674,872	t(4) = 4.426	0.0115

Measurement	Group means	n	Statistical analysis	Effect estimate / 95% CI	Test statistic	Exact *p* value
Mean firing rate (Hz), DIV44–DIV45	Vehicle: 0.2959 → 0.2966; DHA: 0.2955 → 0.5327	5 wells/treatment	Two-way repeated-measures ANOVA	Time × Treatment interaction	F(1,8) = 222.0	<0.01
Network burst duration (s), DIV44–DIV45	Vehicle: 2.456 → 2.550; DHA: 2.447 → 1.847	5 wells/treatment	Two-way repeated-measures ANOVA	Time × Treatment interaction	F(1,8) = 44.92	0.01
Synchrony index, DIV44–DIV45	Vehicle: 0.1175 → 0.1239; DHA: 0.1235 → 0.2247	5 wells/treatment	Two-way repeated-measures ANOVA	Time × Treatment interaction	F(1,8) = 183.2	<0.01
**WT versus SHINE DHA-response analysis (Figure S4)**						
PSD-95 immunofluorescence intensity	WT Veh: 2160; WT DHA: 4133; SHINE Veh: 1081; SHINE DHA: 1816	3 experiments/condition	Two-way repeated-measures ANOVA, matched by experimental run	Genotype × Treatment contrast: −1238; 95% CI −2130 to −345.5. Sidak: WT p = 0.0109; SHINE p = 0.0736	F(1,2) = 35.63	0.01
PSD-95 puncta density	WT Veh: 0.2363; WT DHA: 0.3487; SHINE Veh: 0.09654; SHINE DHA: 0.09838	3 experiments/condition	Two-way repeated-measures ANOVA, matched by experimental run	Genotype × Treatment contrast: −0.1106; 95% CI −0.1796 to −0.04155. Sidak: WT p = 0.0200; SHINE p = 0.9870	F(1,2) = 47.51	0.01
MAP2 intensity	WT Veh: 1259; WT DHA: 1674; SHINE Veh: 446.5; SHINE DHA: 749.6	3 experiments/condition	Two-way repeated-measures ANOVA, matched by experimental run	Genotype × Treatment contrast: −111.9; 95% CI −811.2 to 587.5. Sidak: WT p = 0.1330; SHINE p = 0.2233	F(1,2) = 0.4736	0.5624
Δ Mean firing rate (Hz)	WT Veh: 0.2357; WT DHA: 0.1328; SHINE Veh: 0.000761; SHINE DHA: 0.2372	5 wells/condition	Ordinary two-way ANOVA of DIV45 − DIV44 change scores	Interaction contrast: 0.3393; 95% CI 0.1395 to 0.5392. Sidak: WT p = 0.2643; SHINE p = 0.0054	F(1,16) = 12.96	0.024
Δ Network burst duration (s)	WT Veh: 0.4286; WT DHA: 0.3168; SHINE Veh: 0.09483; SHINE DHA: −0.5997	5 wells/condition	Ordinary two-way ANOVA of DIV45 − DIV44 change scores	Interaction contrast: −0.5827; 95% CI −1.120 to −0.04578. Sidak: WT p = 0.7895; SHINE p = 0.0027	F(1,16) = 5.293	0.0352
Δ Synchrony index	WT Veh: 0.1296; WT DHA: 0.1265; SHINE Veh: 0.006464; SHINE DHA: 0.1012	5 wells/condition	Ordinary two-way ANOVA of DIV45 − DIV44 change scores	Interaction contrast: 0.09786; 95% CI 0.07773 to 0.1180. Sidak: WT p = 0.8758; SHINE p < 0.0001	F(1,16) = 106.2	<0.01
**Exploratory signaling measurements**						

Measurement	Group means	n	Statistical analysis	Effect estimate / 95% CI	Test statistic	Exact *p* value
pERK/tERK ratio	Vehicle: 0.2552; DHA: 0.4371	3 experiments/group	Two-tailed unpaired Student's t-test	DHA − Vehicle: 0.1820; 95% CI −0.09571 to 0.4596	t(4) = 1.819	0.1430
pCREB/tCREB ratio	Vehicle: 1.523; DHA: 3.711	3 experiments/group	Two-tailed unpaired Student's t-test	DHA − Vehicle: 2.188; 95% CI −0.9295 to 5.306	t(4) = 1.949	0.1232

Data represent group means. Immunofluorescence and Western blot analyses were based on three independent experimental runs. Baseline and treatment-related MEA measurements represent replicate wells from a single MEA experiment, with five wells per condition. For longitudinal MEA analyses, the same wells were measured at DIV44 and DIV45. For Figure S4 MEA analyses, Δ represents DIV45 - DIV44. Variability is reported as the 95% confidence interval of the relevant between-group or interaction contrast. Sidak's multiple comparisons test was used following two-way ANOVA where indicated. DHA, docosahexaenoic acid; DIV, day *in vitro*; ERK, extracellular signal-regulated kinase; MAP2, microtubule-associated protein 2; PSD-95, postsynaptic density protein 95; WT, wild type.

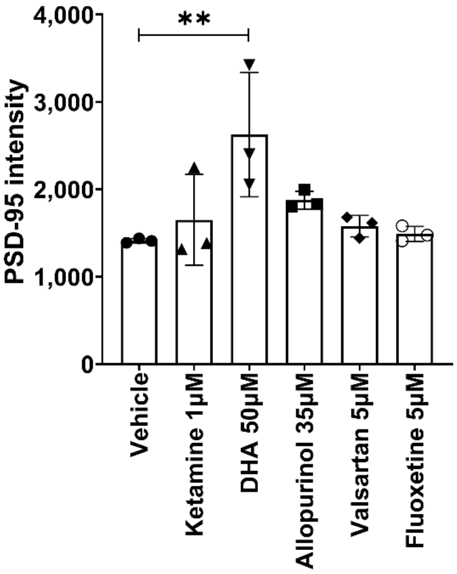

Figure S1. Preliminary targeted screen of selected candidate compounds for effects on PSD-95 in differentiated SH-SY5Y cells. Differentiated SH-SY5Y cells were treated for 24 h with vehicle or the five highest-ranking candidate compounds from an initial 18-compound screen: ketamine (1 µM), DHA (50 µM), allopurinol (35 µM), valsartan (5 µM), and fluoxetine (5 µM). PSD-95 immunofluorescence intensity was used as the screening readout. The figure shows the five highest-ranking candidates based on the magnitude of change relative to vehicle-treated controls. DHA produced the largest increase in PSD-95 immunofluorescence intensity. Data are presented as mean ± SD, with n = 3 independent experiments. Statistical analysis was performed using one-way ANOVA followed by Dunnett's multiple-comparisons test. **$p < 0.01$ vs vehicle.

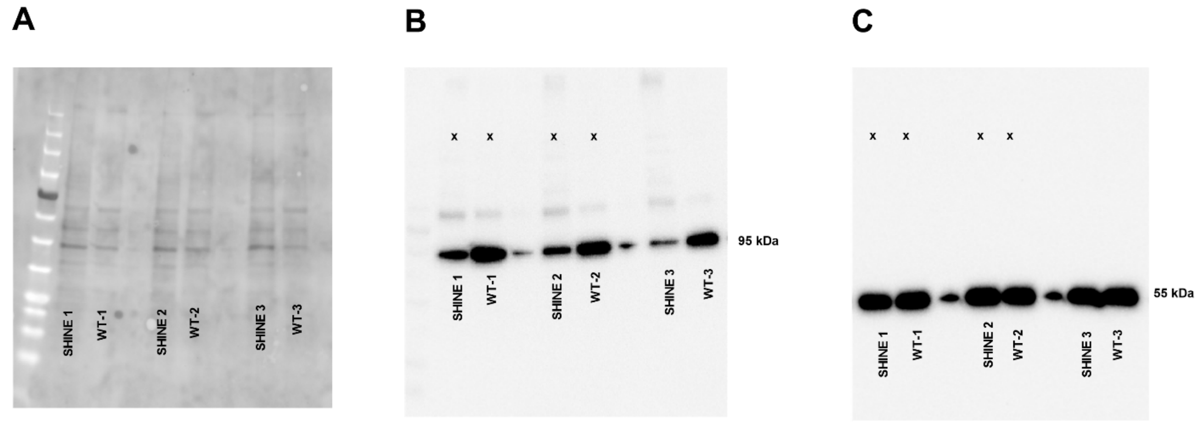

Figure S2. Uncropped Western blot images: WT versus SHINE neurons. **(A)** Stain-Free total-protein image corresponding to the WT and SHINE samples analyzed by Western blotting. **(B)** Uncropped PSD-95 blot (95 kDa). **(C)** Uncropped βIII-tubulin blot (55 kDa). Three independent biological replicates are shown for each condition (n = 3). All samples used for each comparison were run on the same gel. Lanes not included in the representative cropped images presented in the manuscript are indicated with an "X". No lanes were rearranged or spliced.

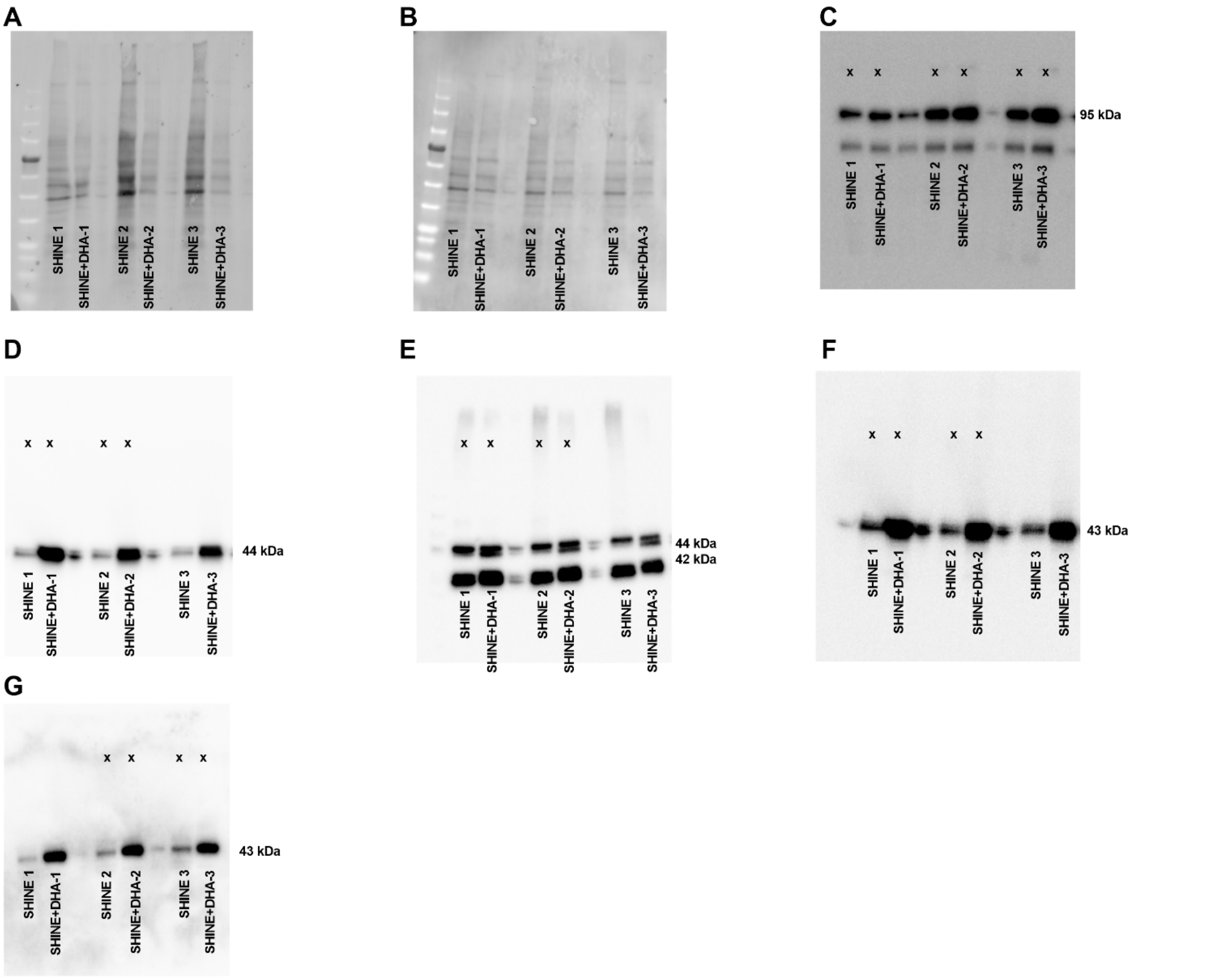

Figure S3. Uncropped Western blot images: SHINE neurons treated with DHA. **(A)** Stain-Free total-protein image corresponding to SHINE vehicle-control and SHINE+DHA samples following 24 h treatment. **(B)** Stain-Free total-protein image corresponding to SHINE vehicle-control and SHINE+DHA samples following 30 min treatment. **(C)** Uncropped PSD-95 blot (95 kDa) following 24 h treatment. **(D)** Uncropped phospho-p44/42 MAPK (pERK1/2; Thr202/Tyr204; 44/42 kDa) blot following 30 min treatment. **(E)** Uncropped total p44/42 MAPK (tERK1/2; 44/42 kDa) blot corresponding to (D). **(F)** Uncropped phospho-CREB (Ser133; 43 kDa) blot following 30 min treatment. **(G)** Uncropped total CREB (43 kDa) blot corresponding to (F). Three independent biological replicates are shown for each condition (n = 3). All samples used for each comparison were run on the same gel. Lanes not included in the representative cropped images presented in the manuscript are indicated with an "X". No lanes were rearranged or spliced.

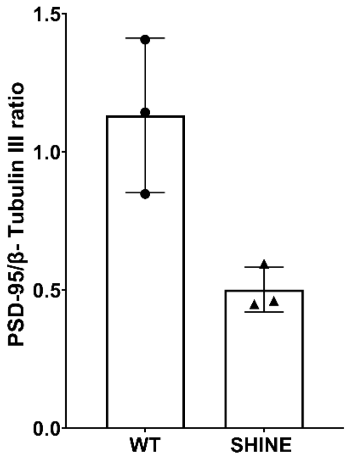

Figure S4. PSD-95 abundance normalized to β-tubulin III in WT and SHINE iPSC-derived neurons. PSD-95 abundance was normalized to β-Tubulin III within each experimental run to assess PSD-95 abundance relative to neuronal content. The PSD-95/β-Tubulin III ratio was lower in SHINE neurons relative to WT neurons, although the difference did not reach statistical significance. Data are presented as mean ± SD, with individual data points representing the three independent experiments (n = 3). Statistical significance was determined using a two-tailed paired Student's t-test.

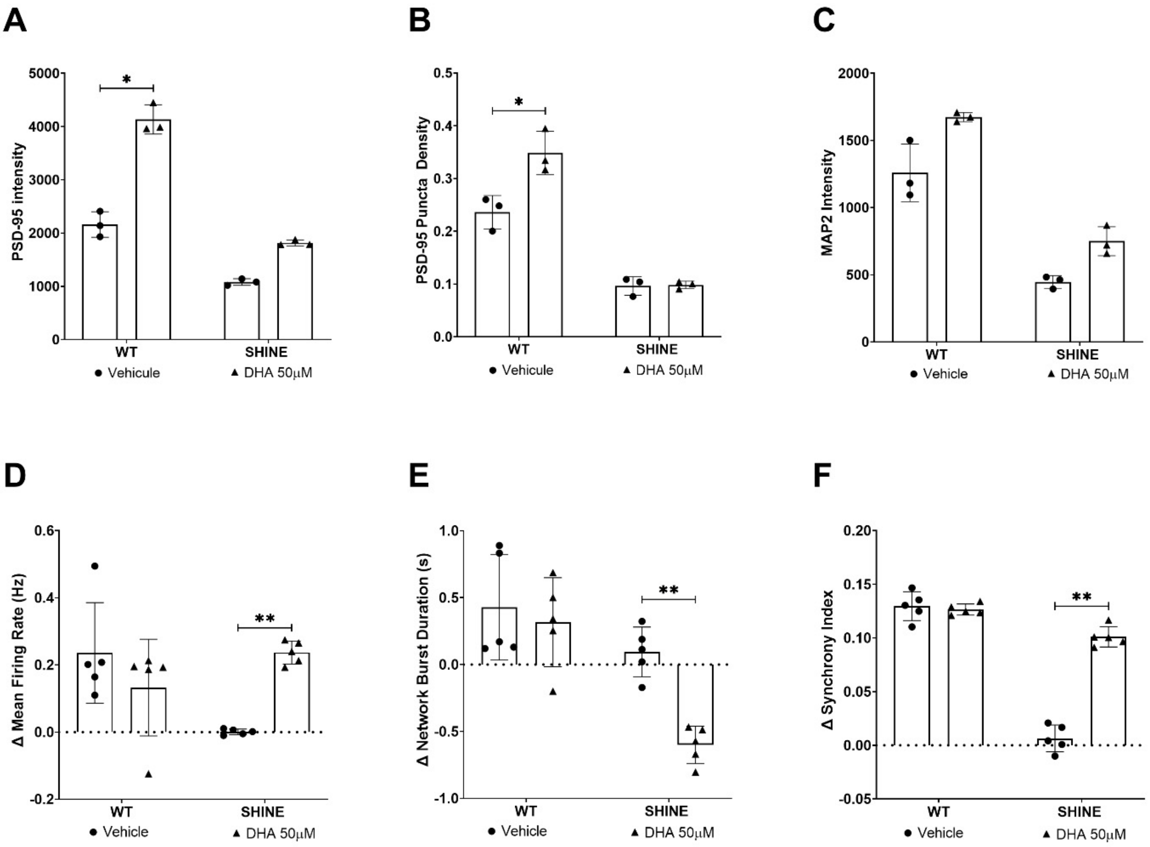

Figure S5. Effects of DHA treatment on synaptic markers and neuronal network activity in WT and SHINE neurons. **(A)** PSD-95 immunofluorescence intensity is increased following DHA treatment in WT neurons, whereas the increase in SHINE neurons is not significant. **(B)** PSD-95 puncta density is increased following DHA treatment in WT neurons, with no significant change in SHINE neurons. **(C)** MAP2 immunofluorescence intensity in WT and SHINE neurons following vehicle or DHA treatment. **(D-F)** Changes from DIV44 to DIV45 (Δ = DIV45 - DIV44) in **(D)** mean firing rate, **(E)** network burst duration, and **(F)** synchrony index. DHA treatment significantly increased mean firing rate and synchrony and reduced network burst duration in SHINE neurons, whereas no significant treatment-related differences were observed in WT neurons. Data are presented as mean ± SD. Individual data points represent three independent experiments in (A-C) and replicate wells from a single MEA experiment in (D-F) (n = 5 wells per condition). Data in (A-C) were analyzed using two-way repeated-measures ANOVA with experimental run as the matched factor, and data in (D-F) using ordinary two-way ANOVA of change scores, followed by Sidak's multiple comparisons test. *$p < 0.05$, **$p < 0.01$.

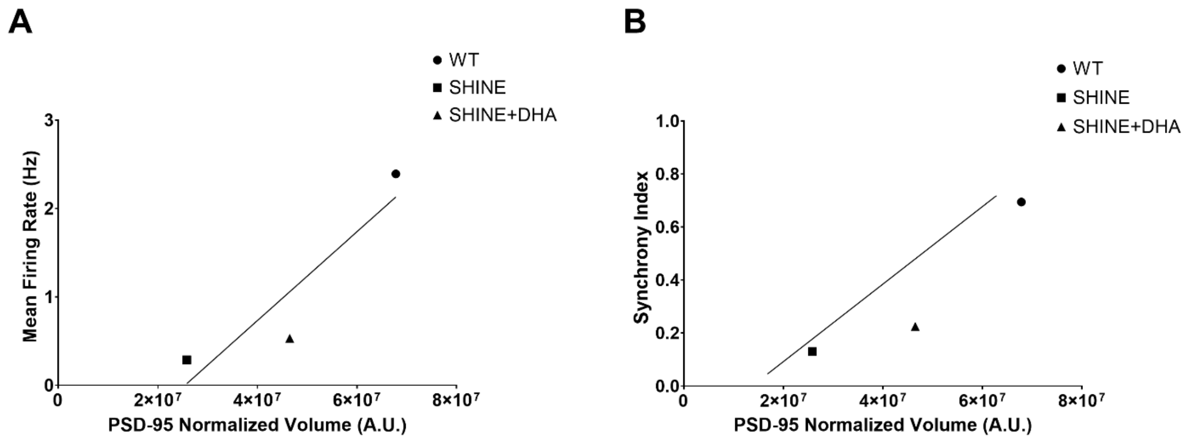

Figure S6. Descriptive relationship between PSD-95 abundance and neuronal network activity across experimental conditions. **(A)** Mean PSD-95 abundance for each experimental condition (WT, SHINE, and SHINE+DHA) plotted against the corresponding mean firing rate. **(B)** Mean PSD-95 abundance plotted against the corresponding mean synchrony index. Each point represents one experimental condition and combines the mean PSD-95 value obtained from three independent Western blot experiments with the corresponding mean MEA value obtained from five neuronal clones. Linear trend lines are shown for descriptive visualization only. Because Western blot and MEA measurements were obtained from separate experiments and were not paired at the individual-sample level, no inferential correlation statistics were performed.
